# Peer-assisted feedback: a successful approach for providing feedback on United States Medical Licensing Exam-style clinical skills exam notes in the United States

**DOI:** 10.3352/jeehp.2019.16.29

**Published:** 2019-10-08

**Authors:** Kira Nagoshi, Zareen Zaidi, Ashleigh Wright, Carolyn Stalvey

**Affiliations:** 1 Buchholz High School, Gainesville, FL, USA; 2 Division of General Internal Medicine, Department of Medicine, University of Florida, Gainesville, FL, USA; Hallym University, Korea

**Keywords:** Clinical competence, Feedback, Medical students, Peer group, United States

## Abstract

**Purpose:**

Peer-assisted learning (PAL) promotes the development of communication, facilitates improvements in clinical skills, and is a way to provide feedback to learners. We utilized PAL as a conceptual framework to explore the feasibility of peer-assisted feedback (PAF) to improve note-writing skills without requiring faculty time. The aim was to assess whether PAL was a successful method to provide feedback on the United States Medical Licensing Exams (USMLE)-style clinical skills exam notes by using student feedback on a survey in the United States.

**Methods:**

The University of Florida College of Medicine administers clinical skills examination (CSEs) that include USMLE-like note-writing. PAL, in which students support the learning of their peers, was utilized as an alternative to faculty feedback. Second-year (MS2) and third-year (MS3) medical students taking CSEs participated in faculty-run note-grading sessions immediately after testing, which included explanations of grading rubrics and the feedback process. Students graded an anonymized peer’s notes. The graded material was then forwarded anonymously to its student author to review. Students were surveyed on their perceived ability to provide feedback and the benefits derived from PAF using a Likert scale (1–6) and open-ended comments during the 2017–2018 academic year.

**Results:**

Students felt generally positively about the activity, with mean scores for items related to educational value of 4.49 for MS2s and 5.11 for MS3s (out of 6). MS3s perceived peer feedback as constructive, felt that evaluating each other’s notes was beneficial, and felt that the exercise would improve their future notes. While still positive, MS2 students gave lower scores than the MS3 students.

**Conclusion:**

PAF was a successful method of providing feedback on student CSE notes, especially for MS3s. MS2s commented that although they learned during the process, they might be more invested in improving their note-writing as they approach their own USMLE exam.

## Introduction

### Background

In the United States, medical students are required to take the United States Medical Licensing Exams (USMLE), including the Step 2 Clinical Skills (CS) exam given to fourth-year medical students. This exam assesses students’ ability to perform history-taking and physical examinations, communicate appropriately with patients using standardized patients, and write effective notes [1].

The University of Florida College of Medicine (UFCOM) administers biannual clinical skills examinations (CSEs) to evaluate student progress in the curriculum and to help them prepare for the USMLE Step 2 CS exam. The CSEs include note-writing stations mimicking the Step 2 CS notes format. Providing effective note feedback was challenging due to the small number of faculty graders. As part of a quality assurance process for medical education, the CSE feedback process was reviewed [2]. Student notes were initially evaluated by faculty members who assigned a numeric grade with few narrative comments. The magnitude of the workload and the limited number of faculty caused delays in feedback. In addition, the feedback was of limited utility to students, diminishing the effectiveness of CSEs as a preparatory tool. Therefore, UFCOM sought to find alternative methods of reviewing CSE notes to improve the feedback process.

Peer-assisted learning (PAL) is a collaborative education method in which students share information with and support the learning of their peers [3]. The benefits of PAL also include further development of communication skills and facilitation of skill acquisition and improvement; therefore, PAL improves clinical skills training and the manner in which feedback is presented to learners [4].

### Purpose

As a part of our quality assurance process, we utilized PAL as a conceptual framework to explore the feasibility of peer-assisted feedback (PAF) because UFCOM sought to find alternative methods of reviewing CSE notes to improve the feedback process. The study aimed to evaluate whether PAF on CSE notes could provide constructive, beneficial feedback that would help students improve note-writing in the future from the students’ perspective. Specifically, the students were asked about their comfort in giving feedback to peers and receiving feedback from peers, as well as their perceptions regarding the educational value of the activity overall.

## Methods

### Ethics statement

This study was approved by the University of Florida Institutional Review Board (IRB201900031) as an exempt study because it was done as part of the routine educational curriculum. It was conducted during the 2017–2018 academic year at UFCOM in Gainesville, Florida.

### Study design

This study involved both a descriptive analysis based on the survey results and a qualitative analysis of students’ comments.

### Setting/participants

The educational assessment program at UFCOM incorporates CSEs at the end of each semester during the first 3 years. Most CSEs consist of 6–11 stations utilizing standardized patients to complete case checklists. The participants in the study intervention included 135 second-year students (MS2s) who took the end-of-second-year CSE (CSE 2) and 132 third-year students (MS3) who took the end-of-third-year CSE (CSE 3). The CSEs selected for the intervention were based on convenience sampling.

As part of the CSE, students write USMLE Step 2 CS-style notes on 2–3 encounters at computer stations outside of the exam rooms. Note-grading sessions were held immediately following the conclusion of the year-specific CSE. During the session, the Medical Director of the Assessment Center conducted a detailed session on note-grading, explaining detailed grading rubrics and how to provide narrative feedback. The training sessions lasted approximately ninety minutes and consisted of a PowerPoint lecture with sample notes and feedback. Students were given 2 anonymized peers’ notes from the exam in a random fashion. Students used the rubric shown in Supplement 1 to evaluate the notes with space to include written feedback to peers. The faculty leader walked around the room, observing the graded rubrics and written feedback, giving guidance to the student graders as needed, and answering questions. Afterwards, the notes with completed graded rubrics were returned to the faculty, who returned them anonymously to the student authors. Students were given time to review their peer-graded notes.

### Survey process

Following completion of the activity, 261 students completed a survey that assessed their reactions to the session, including a self-assessment of their ability to evaluate peers’ clinical reasoning process displayed in the CSE note and the educational value of the peer review process. The 9-item survey utilized a 6-point Likert scale in which only the endpoints were labelled: 1=strongly disagree and 6=strongly agree (Table 1). The survey included 2 open-ended questions asking students to describe what was done well in the session and to provide suggestions for improvement.

### Validity and reliability of the survey tool

**Fig. 1.**
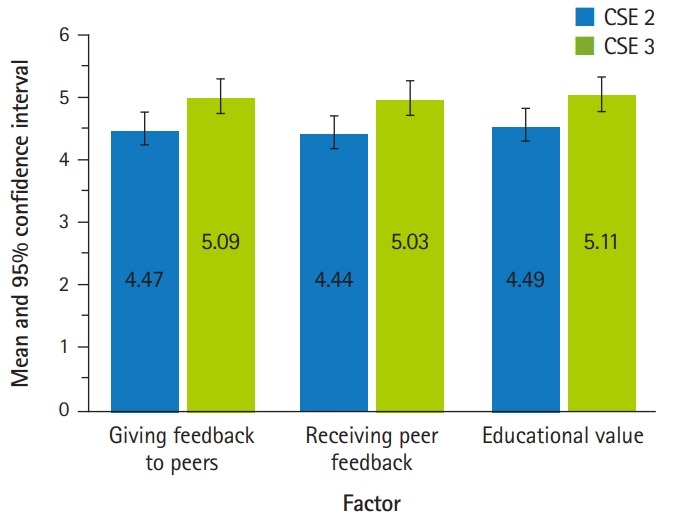
Students’ perception of peer-assisted learning sessions: CSE 2 versus CSE 3. CSE, clinical skills examination.

Construction of the survey questions was guided by the literature and by consultation with medical education experts at UFCOM; therefore, content validity was fulfilled. As for construct validity, responses to the 9 survey items were subjected to exploratory factor analysis using squared multiple correlations as communality estimates. The independent-sample t-test was used for comparisons between groups. All statistical analyses were conducted using SAS ver. 9.4 (SAS Institute Inc., Cary, NC, USA). Graphs were constructed using IBM SPSS ver. 25.0 (IBM Corp., Armonk, NY, USA). The principal factor method was used to extract the factors, followed by oblique rotation to aid interpretability using the Promax method. The screen test suggested 3 meaningful factors, and these factors were retained for rotation. In interpreting the rotated factor pattern (Table 2), an item was said to load on a given factor if the factor loading was 0.50 or greater for that factor. Using these criteria, groups of 3 items were found to load on each of the 3 factors. Items 1, 2, and 3 were found to load on the first factor, which was labeled “giving feedback to peers.” Items 4, 5, and 6 loaded on the second factor, labeled “peer feedback received.” Items 7, 8, and 9 loaded on the third factor, labeled “educational value” of the peer feedback session. Using the oblique rotation method allows factors to be correlated. The correlation between factor 1 and factor 2 was r=0.37, the correlation between factor 1 and factor 3 was r=0.47, and the correlation between factor 2 and factor 3 was r=0.65. These 3 factors accounted for 72% of the variance in student responses. Additionally, a non-refined method was used to compute factor scores, in which student responses were averaged for each of the 3 factors (subscales) described. The mean factor scores for the 2 groups and the 95% confidence interval (CI) for the mean are included in Table 2 and depicted in Fig. 1. The Cronbach α coefficient was also computed to measure the internal consistency of the survey items common to both groups in the aggregate and for each group. Table 1 provides descriptive statistics for the survey items for all participants and by CSE level. For the aggregated data, the internal consistency of the survey items, as measured by the Cronbach α coefficient, was 0.89; for CSE2, it was 0.88 and for CSE3, 0.86.

### Qualitative analysis

Students’ written comments were analyzed. Utilizing the qualitative methodology of Braun and Clarke [5], an inductive thematic analysis was performed following the 6 phases of analysis. KN independently analyzed the data iteratively and identified themes until saturation was obtained, focusing on the patterns and richness of responses rather than the number of responses, and assigned comments to themes. ZZ reviewed the themes for accuracy and an audit trail was maintained with comments. Study participants were invited to review the thematic analysis to verify the accuracy of themes. Quality criteria in qualitative research were used to assess the content validity and reliability of the data [6]. Member checking was done by asking participants to provide feedback on the interpretation of the data. A detailed description of the context of the study was been provided to increase transferability (i.e., the extent to which the findings can be applied to different settings).

### Statistical methods

Descriptive and comparative statistics of the survey results were calculated using IBM SPSS ver. 25.0 (IBM Corp.).

## Results

At the end of the exercise, students completed a survey (Table 1), with a response rate of 261 of 267 (97.7%) (Dataset 1). Descriptive statistics for the survey items were computed.

### Quantitative study results

The mean score for the survey items self-assessing the ability to give feedback was 4.47 (95% CI, 4.31–4.52) for MS2s and 5.09 (95% CI, 4.96–5.21) for MS3s. For the items addressing the benefits of peer evaluation, the mean score was 4.44 (95% CI, 4.25–4.63) for MS2s and 5.03 (95% CI, 4.89–5.18) for MS3s. The mean score for the items related to educational value was 4.49 (95% CI, 4.29–4.68) for MS2s and 5.11 (95% CI, 4.97–5.26) for MS3s. Given the 1–6 scale, these findings show that students felt generally positively about this session; however, comparing the subscale scores between the 2 groups with the independent-samples t-test showed that MS3s were significantly more positive than MS2s (P<0.0001 for all comparisons).

Table 1 displays that MS3s had a greater appreciation for the PAF intervention. MS3s were more confident regarding their ability to provide feedback to peers, but both groups wanted more training on delivering feedback. MS3s valued the feedback received from peers more then MS2s, particularly regarding error identification. They perceived that the feedback was constructive and that they learned from reading the notes. More MS3s felt that the exercise would improve their future notes.

### Qualitative study results

Thematic analysis of the open-ended comments revealed 2 major themes: “Helpful aspects of the intervention” and “areas of concern.” Table 3 provides a list of the subthemes. Regarding “helpful aspects of the intervention,” many students appreciated having a checklist for each note, emphasizing the opportunity to review notes with a checklist helped them “understand expectations” and “identify gaps”:

“I think the note checklist was helpful because it made me realize which things I was missing.” (MS2-05)
“Examples of acceptable and unacceptable, the specific, itemized checklists were very helpful as a learning tool. And also made the peer grading process very efficient.” (MS3-07)

Students commented that the intervention helped “demystify the Step 2 CS.” It provided “good practice,” “insights,” and “explanation of what is required for the CS exam.” The majority of students noted that reviewing peer notes was valuable.

“I was able to note how my peer did things differently than I did and how I can incorporate that into my future notes.” (MS3-09)

Regarding areas of concern, some students noted that they did not feel confident providing feedback to others:

“After making all the mistakes that I made I didn’t feel justified to provide peer feedback.” (MS2-01)

Additionally, a few students commented that the feedback they received was inadequate.

“I think just making sure everyone gives feedback comments and constructive suggestions is important. I didn’t get any and would have liked to see how I can further improve my note writing.” (MS3-12)

While students appreciated the opportunity to understand expectations for Step 2 CS, they noted they would likely benefit from repetition of sessions closer to when they will prepare for the USMLE CS exam. Students’ responses regarding the timing and duration of the session were variable. The MS3s appreciated that the session was close to their USMLE CS exam, while the MS2s felt they were too far placed from the USMLE CS exam to consider the intervention helpful. One MS2 student commented that the session could be improved by “Not doing it now. Step 2 [is] 2 years away.” Regarding the duration of the intervention, while some students appreciated that the intervention was timed so that they were “going back through the encounters while they were fresh,” others commented that it was “a very long day and we might benefit more from this being on a different day.”

## Discussion

### Key results

This study has 2 main findings. First, a PAF intervention to provide feedback after CSEs was received favorably by students. Second, the appreciation of PAF among medical students significantly improved with progression through medical school.

### Interpretation and suggestions

All participants found the peer feedback to be useful and agreed that they learned by reading and providing feedback to peers, but MS3s found the intervention more beneficial then MS2s. The MS2s were less confident in their skills to provide feedback and make judgments on peer’s notes. These results corroborate findings by Burgess et al. [7], where students commented in focus group discussions that they were more comfortable receiving peer feedback in their fourth year than in their third year because they found the feedback to be more accurate and their peers more knowledgeable.

One of the possible reasons that MS2s felt less benefit from the intervention is that the greater medical knowledge of the MS3s compared to the MS2s could have made the MS3s feel more confident in their clinical skills and, in turn, their ability to grade a peer’s note. It is also possible that greater familiarity with the feedback process influenced the responses. While they may not have activities specifically intended to train them in providing feedback, as students’ progress through medical school, they often participate in activities that require them to give peers feedback on history-taking, physical exams, and presentation skills. It is possible, with more experience, students become more comfortable in giving feedback. This does not negate the utility of sessions specifically dedicated to teaching students how to receive and provide feedback, even though others have shown the futility of a single “teaching the skill of feedback session [8].”

It is important to plan sessions longitudinally and iteratively using different forms of feedback delivery. At our institution, as students sit for the CSE repeatedly over the years, they are also repeatedly participating in the peer grading session with the Medical Director of the Assessment Center. We hope to gather further data over time to see whether the repeated sessions impact students’ confidence in giving feedback. Henderson et al. [9] noted that such continuing learning sessions supporting pre-clinical students to identify, practice, and reflect on communication skills can be useful.

This study also demonstrates how testing drives learning. MS2s appeared to be less motivated to improve their note-taking because the USMLE Step 2 CS would be taken later and was not an immediate concern. Students commented that the session helped them prepare for the USMLE Step 2 CS exam, and reviewing their peers’ notes provided them with insights on how to improve their performance. In the future, we hope to draw students’ attention to how to improve giving and receiving feedback, in addition to performance improvements.

Our study shows that PAF can be successfully used to enhance learning and help students prepare for high-stakes exams. In order for PAF to be effective, we recommend following the PAL framework developed by Ross and Cameron [10]. The PAL planning framework facilitates the generation of a robust PAL plan that takes into account the existing literature, as well as common pitfalls and local context. Like PAL, those planning to undertake PAF interventions need to reflect on students’ prior experience with giving feedback and additional training/preparation they may need. PAL tutors need feedback to enable them to reflect upon and develop their own teaching abilities. Therefore, we recommend dedicated sessions on teaching students how to give and receive feedback.

### Limitations

We report the initial results from a PAF intervention over a 1-year period. There were several limitations of this study. Further data collection is needed to establish long-term acceptability and benefit. We hope to further structure the instructional session by providing students a video and interactive PowerPoint on giving and receiving feedback to help students prepare for the session and feel more comfortable with the activity. Additionally, future changes will be made to the survey, as only the endpoints of the Likert scale were labeled. This could have introduced bias as students subjectively and individually interpreted the meanings of the intermediate scores. Finally, because the UFCOM pass rate of the USMLE Step 2 CS exam is already high and the scores are not quantitatively reported (pass/fail only), it is difficult to objectively assess the efficacy of this intervention by analyzing Step 2 CS improvements.

### Conclusion

In an era where faculty struggle with balancing clinical revenue, teaching, and service, PAF is an educational framework that reduces faculty workload. If thoughtfully planned, it can help with student learning and can improve students’ ability to give and receive feedback.

## Figures and Tables

**Table 1.** Survey items and descriptive statistics by group

Question	CSE 2			CSE 3		
	N	Mean±SD	95% CI	N	Mean±SD	95% CI
Q1: I possess adequate knowledge & skills to provide feedback to my peers.	133	4.61±0.89	4.46–4.76	127	5.16±0.69	5.04–5.28
Q2: I felt confident in making a judgment on a peer’s post-encounter note.	133	4.36±1.00	4.19–4.53	127	5.06±0.74	4.93–5.19
Q3: I felt confident in providing feedback to my peer(s).	132	4.42±0.99	4.25–4.59	127	5.04±0.78	4.90–5.18
Q4: I received useful peer feedback on my notes.	132	4.29±1.26	4.07–4.51	127	4.88±0.99	4.71–5.05
Q5: The feedback I received identified errors and/or missing information in my notes.	133	4.78±1.11	4.59–4.97	127	5.15±0.99	4.98–5.32
Q6: Peer feedback provided constructive suggestions for improving my notes.	133	4.23±1.39	3.99–4.47	126	5.06±0.94	4.90–5.22
Q7: This feedback session will help me write better notes in the future.	133	4.57±1.19	4.37–4.77	127	5.13±0.95	4.96–5.30
Q8: I learned by reading and providing feedback on another student’s notes.	133	4.60±1.28	4.38–4.82	127	5.17±0.94	5.01–5.33
Q9: Providing feedback on the notes was a useful learning activity.	121	4.23±1.23	4.01–4.45	119	4.92±1.09	4.72–5.12

CSE, clinical skills examination; SD, standard deviation; CI, confidence interval

**Table 2.** Factor loadings and factor scores by group

Item	Rotated factor pattern			Mean (95% confidence interval) factor score								
				Giving feedback to peers (factor 1)			Receiving feedback from peers (factor 2)			Educational value (factor 3)		
	Factor 1	Factor 2	Factor 3	MS2	MS3	t (P)-value	MS2	MS3	t (P)-value	MS2	MS3	t (P)-value
Q1	0.81^*^	0.2	0.22	4.47 (4.31–4.62)	5.09 (4.96–5.21)	6.13 (<0.0001)						
Q2	0.91^*^	0.19	0.19									
Q3	0.91^*^	0.12	0.21									
Q4	0.16	0.79^*^	0.34				4.44 (4.25–4.63)	5.03 (4.89–5.18)	4.88 (<0.0001)			
Q5	0.22	0.58^*^	0.15									
Q6	0.08	0.79^*^	0.37									
Q7	0.17	0.43	0.67^*^							4.49 (4.29–4.68)	5.11 (4.97–5.26)	5.07 (<0.0001)
Q8	0.31	0.28	0.73^*^									
Q9	0.19	0.25	0.71^*^									

* P<0.05.

**Table 3.** Thematic analysis of open-ended comments

Theme	Subthemes
Helpful aspects of the intervention	Provision of checklist
	Discussion of expectations
	Demystifying USMLE Step 2 CS
	Opportunity to review peers’ work
Areas of concern	Feedback provided was not adequate
	Timing of the session
	Confidence in ability to provide feedback
	Preference for faculty or senior peers
	Need for refresher sessions prior to USMLE Step 2 CS

USMLE, United States Medical Licensing Exams; CS, Clinical Skills.

## Footnotes

**Data availability** Data files are available from Harvard Dataverse: https://doi.org/10.7910/DVN/EOC6OC Dataset 1. Final inputted data.

## Supplementary materials

Supplementary materials are available from Harvard Dataverse: https://doi.org/10.7910/DVN/EOC6OC

Supplement 1. Example of a grading rubric used during a peer-assisted learning session.

Supplement 2. Audio recording of the abstract.
