# Supplementary material for: Peer-assisted feedback: a successful approach for providing feedback on United States Medical Licensing Exam-style clinical skills exam notes in the United States
Source: J Educ Eval Health Prof. 2019 Oct 8;16:29. doi: 10.3352/jeehp.2019.16.29 (PMC6817788; doi:10.3352/jeehp.2019.16.29)
Supplement: Supplementary file 1 — Supplement 1. Example of a grading rubric used during a peer-assisted learning session. [file jeehp-16-29-suppl.pdf]

J Educ Eval Health Prof 2019;16:29 • https://doi.org/10.3352/jeehp.2019.16.29

Supplement 1. Example of a grading rubric used during a peer-assisted learning session

		Done	Not Done	
LOCATES	Age			Possible Comments:<br>Be careful with abbreviations,<br>Could use less words (and characters) to save time,<br>Missing LOCATES items,<br>Missing history items showing you are ruling in and out various diagnoses,<br>A lot of spelling mistakes...
	Gender			
	Onset			
	Describe palpitations - heart racing, what it felt like			
	Duration of palpation events			
	Frequency of events			
	Aggravating factors			
	Alleviating factors			
ROS/DDX	Chest pain			
	SOB			
	Lightheaded/Dizzy			
	Stress, anxiety, depression, insomnia			
	Caffeine			
	Supplement use			
	Drug use			
	Weight loss			
	More frequent bowel movements			
	Change in menses			
	Temperature intolerance			
	PMH			
	PSH			
	Social Hx			
	Meds			
	Family Hx, FHx thyroid/cardiac			
Physical Exam		Done	Not Done	
	General Statement			Possible Comments:<br>Abbreviations,<br>More specific physical exam findings,<br>Included less pertinent physical exam sections and could have saved time by not including them,<br>Could have included...
	BP			
	Heart Rate			
	Temp			
	Thyroid			
	S1, S2 normal			
	regular rate and rhythm			
	no murmurs, rubs or gallops, no S3, S4			
	Pulses			
	Reflexes			

Differential Dx		BEST	ACCEPTABLE	NOT ACCEPTABLE/ not on list	LEFT BLANK
	Ddx #1 on list?				
	Number of items supporting it				

Ddx #2 on list?				

Number of items supporting it				

Ddx #3 on list?				
Number of items supporting it				

Differential Diagnoses:
Best Diagnoses: Stimulant drink additives, Graves' disease, hyperthyroid, early Hashimoto's, toxic multinodular goiter, cardiac arrhythmia, SVT/supraventricular tachycardia, a-fib/flutter

Other Acceptable Dx: Graves' disease, hyperthyroid, early Hashimoto's, toxic multinodular goiter, anxiety, cardiac arrhythmia, SVT/supraventricular tachycardia, a-fib/flutter, drug use/illegal drugs/stimulants, pheochromocytoma

NOT Acceptable Dx: Menopause/hot flashes, thyroid disease (not specific enough)
